# Reactive oxygen species mediate heat stress-induced apoptosis via ERK dephosphorylation and Bcl-2 ubiquitination in human umbilical vein endothelial cells

**DOI:** 10.18632/oncotarget.14186

**Published:** 2016-12-25

**Authors:** Li Li, Hongping Tan, Hong Yang, Feng Li, Xuan He, Zhengtao Gu, Ming Zhao, Lei Su

**Affiliations:** ^1^ Department of Intensive Care Unit, The Third Affiliated Hospital of Southern Medical University, Guangzhou 510630, P.R. China; ^2^ Department of Pathophysiology, Southern Medical University, Guangdong Provincial Key Laboratory of Shock and Microcirculation Research, Guangzhou 510515, P.R. China; ^3^ Department of Epilepsy Surgery, Guangdong Sanjiu Brain Hospital, Guangzhou 510510, P.R. China; ^4^ Southern Medical University, Guangzhou, 510515, China; ^5^ Department of Pathophysiology, Southern Medical University, Guangdong Provincial Key Laboratory of Shock and Microcirculation Research, Guangzhou 510515, P.R. China; ^6^ Department of Intensive Care Unit, Guangzhou General Hospital of Guangzhou Military Command, Guangzhou, P.R. China

**Keywords:** heat stress, reactive oxygen species, apoptosis, ERK, Bcl-2

## Abstract

Heat stress can induce the mitochondrial apoptotic pathway in HUVEC cells, indicating that apoptosis may be a prominent pathological feature of heat stroke, however, little is known about the precise mechani sms involved in it. In this study, we describe the apoptotic effect of intense heat stress on HUVEC cells and our investigation of its underlying mechanisms. Treatment of cells with intense heat stress induced production of reactive oxygen species (ROS) and a concomitant increase in activation of the mitochondrial apoptotic pathway. Furthermore, by over-expression of MnSOD and GPx in cells, we show that ROS, and especially superoxide, is the primary oxidative species induced by intense heat stress and responsible for cell death. In addition, we explored the mechanism by which superoxide regulates the apoptotic effect of intense heat stress, and found that it involved Bcl-2 down-regulation through ubiquitin - proteasomal degradation. Superoxide production also led to Bcl-2 dephosphorylation through inactivation of MAP kinase ERK1/2, which promoted Bcl-2 ubiquitination. Taken together, these findings describe a novel pathway downstream of heat stress-induced apoptosis in HUVEC cells, and provide new insight into the process of redox-mediated down-regulation of Bcl-2 and apoptosis induction. These results could be important in the understanding of pathogenesis of heat stroke and for the development of preventive and treatment measures, both of which are currently lacking.

## INTRODUCTION

Heat stroke is a severely life-threatening heat-related illness, characterized by a rapid rise in core body temperature to greater than 40°C, and central nervous system dysfunction, such as delirium, convulsions, or coma [1–3]. Environmental heat exposure is a serious cause of natural death worldwide, and is responsible for at least 7% of wilderness-related deaths [4]. However, there is limited understanding of the mechanisms which mediate morbidity and mortality during heat stroke.

Accumulating evidence proves that high heat can stimulate cell death and tissue injury, and that apoptosis plays a key role during this process. Both *in vitro* and *in vivo* studies have demonstrated that elevated temperatures can result in direct injury to vascular endothelium, indicating that targeted endothelial cell damage may be the underlying cause of prominent heatstroke features [5–8]. Furthermore, it has been observed that acute heat stress-induced endothelial cell damage results in apoptosis [4, 9], suggesting that apoptotic death of endothelial cells might be a critical event in the pathogenesis of heat stroke. In light of these findings, the molecular mechanisms of endothelial cell apoptosis induced by heat stress require further study.

Our recent work showed that heat stress induces the mitochondrial apoptotic pathway in HUVEC cells, with ROS acting as an upstream participant in this process [10–12]. It has also been confirmed that over-expression Bcl-2 in HUVEC cells significantly decreases intense heat stress-induced apoptosis, the loss of *ΔΨ*m and cytochrome c release [10–12]. Thus, down-regulation of Bcl-2 by intense heat stress may also be an apoptotic trigger in HUVEC cells, although we did not observe this in our previous study. It has been shown that expression and stability of the Bcl-2 protein can be mediated by different reactive species, including superoxide (O_2_^.-^), hydrogen peroxide (H_2_O_2_) and nitric oxide (NO), by phosphorylation, degradation, dimerization, transcription, and posttranslational modification [13]. Even though evidence has implicated ROS in regulation of apoptosis by mediating Bcl-2 expression through ubiquitin–mediated proteasome degradation, the precise mechanisms of and specific ROS involved have not been investigated [14–17]. Moreover, as Bcl-2 phosphorylation is strongly associated with apoptosis and de-phosphorylation is an essential step for Bcl-2 ubiquitination [14–17], we sought to identify the major phosphorylation signaling cascades responsible for Bcl-2 de-phosphorylation and ubiquitination in response to intense heat stress.

The overall objective of this study was to identify the specific ROS involved in and the underlying mechanisms of intense heat-induced apoptosis in HUVEC cells. We have observed that superoxide is an upstream mediator of intense heat stress-induced apoptosis and exerts its pro-apoptotic effect by activating the ubiquitin–proteasome and mitochondrial apoptotic pathways in HUVEC cells. Finally, we further elucidated the role of the MAP kinase ERK1/2 pathway in Bcl-2 de-phosphorylation and ubiquitin–mediated proteasome degradation. Our results in HUVEC cells lay the foundation for detailed study of molecular mechanisms of apoptosis initiation and regulation in the endothelial system in response to heat stroke.

## RESULTS

### Effects of intense heat stress on HUVEC cell viability and apoptosis

**Figure 1:**
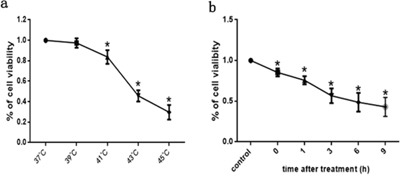
Heat stress reduced cell viability in HUVEC cells **a**. Cells were exposed to the indicated temperature for 2h (37°C, 39°C, 41°C, 43°C or 45°C), and were further incubated at 37°C for 6h. The percentages of viability were assessed by CCK-8. **b**. Cells underwent intense heat stress (43°C) for 2h, and were further incubated at 37°C for the indicated times (0h, 1h, 3h, 6h, or 9h). The percentages of viability were assessed by CCK-8. The data shown represent the mean ±SD of three independent experiments, performed in triplicate. *P < 0.05, statistically significant relative to control.

CCK-8 assays were employed to investigate changes in viability following heat stress in HUVEC cells. As shown in Figure 1a, cells were maintained in standard culture media for 48 h at 37°C prior to a temperature shift to 39°C, 41°C, 43°C, or 45°C for the duration of heat stress treatments. Cell viability declined drastically after cells were cultured at elevated temperatures, as indicated by the temperature-dependent reduction in formazan formation. In Figure 1b, cells were harvested at 0,1,3,6 and 9h after 2h of heat stress (43°C), and cell viability declined significantly after heat stress in a time-dependent manner. These results suggest that heat stress exerts a cytotoxic effect on HUVEC cells.

### ROS involved in the mitochondrial apoptotic pathway is induced by intense heat stress in HUVEC cells

**Figure 2:**
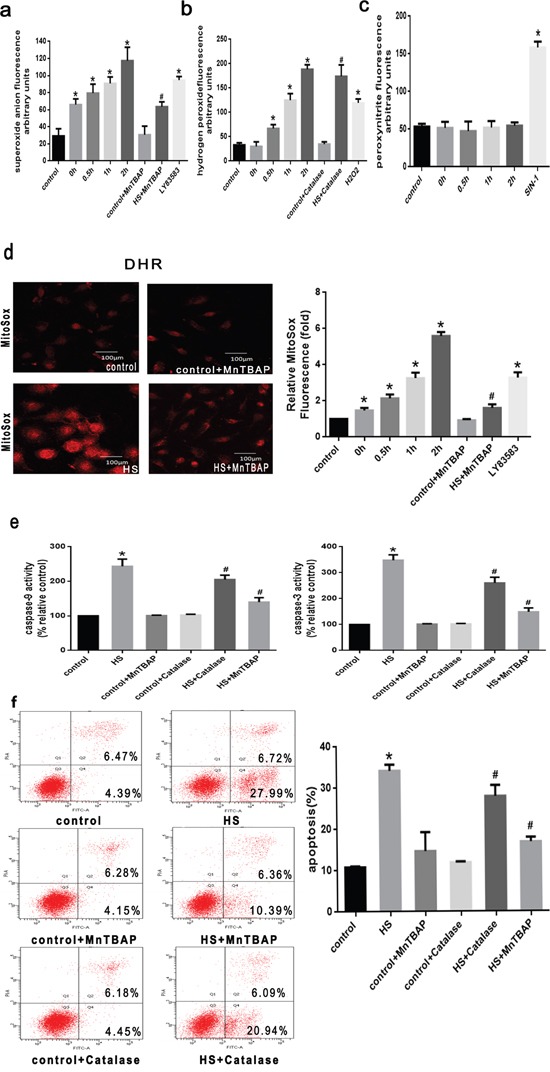
ROS involved in the mitochondrial apoptotic pathway is induced by intense heat stress in HUVEC cells **a**. O_2_^.-^ production was measured with a commercial superoxide anion assay kit based on the oxidation of luminal, LY83583 (10μM) were used as positive control. **b**. H2O2 production was measured with peroxyfluor-6 acetoxymethyl ester (PF6-AM), H2O2 (25μM) were used as positive control. **c**. ONOO- was measured with luminol-amplified chemiluminescence, SIN-1(1mM) were used as positive control. **d**. Mitochondrial superoxide were labeled with MitoSOX™ Red, and Mitochondrial superoxide generation was labeled by laser scanning confocal microscopy. **e-f**. Cells were pretreated with MnTBAP (100μM) or Catalase (1000 U/μl) for 0.5h prior to heat stress (43°C) for 2h, and further incubated at 37°C for 6h. Enzymatic activity of caspase-9 and-3 was measured in cell lysates using the fluorogenic substrates Ac-LEHD-AFC and Ac-DEVD-AMC, respectively, and caspase activity was expressed relative to the control. Apoptosis induction was analyzed by flow cytometry using Annexin V-FITC/PI staining. Each value represents the mean ± SD of three separate experiments, *P < 0.05, relative to the control group (37°C), ^#^*P* < 0.05, as compared to heat stress group (43°C).

Given that ROS generation plays an important role in heat stress [11, 18, 19], we first quantified the induction of cellular ROS production in HUVEC cells exposed to intense heat stress, by flow cytometry using the fluorescent probes DHE and DHR, which detect O_2_^.-^and H_2_O_2_, respectively. As shown in Figure 2a, 2b, O_2_^.-^ levels noticeably increased immediately after heat stress (0h), and the chemiluminescence signal was amplified by LY83583 (O2-donor) and inhibited by the addition of the SOD mimetic MnTBAP (O_2_^.-^ scavenger) after heat stress (1h). H2O2 levels climbed significantly at 0.5h after heat stress, correlating with elevated PF6-AM signal, whereas catalase, an H2O2 scavenger, led to decreased PF6-AM. Thus, time-dependent heat stress results in elevation of both O_2_^.-^ and H_2_O_2_, levels, with the increase in O_2_^.-^ preceding the increase in H_2_O_2_. We also used mitochondria-targeted hydroethidium (MitoSOX™ Red) to explore mitochondria as a potential source of superoxide generation. As seen in Figure 2c, MitoSOX fluorescence intensity exhibited a similar increasing trend for the generation of O_2_^.-^ after heat stress, and was inhibited by addition of MnTBAP (O_2_^.-^ scavenger) after heat stress (1h), supporting the generation of mitochondrial superoxide induced by heat stress.

Our previous studies have revealed that heat stress lead to activation of caspase-9,-3 and induced apoptosis, whereas caspase-4 and caspase-8 were not activated, indicating that heat stress triggers mitochondrial apoptotic pathways in HUVEC cells [11, 12]. These results indicate that heat stress first induces an increase in O_2_^.-^, levels, and so we next tested whether O_2_^.-^ is a key mediator of apoptosis induced by intense heat stress. Cells were treated with intense heat stress in the presence or absence of O_2_^.-^ and H_2_O_2_ scavenger molecules. As shown in Figure 2d and 2e, both MnTBAP and Catalase inhibited caspase-9,-3 and apoptosis after intense heat stress (6h). However, the potent inhibitory effect of MnTBAP further indicates that superoxide plays an important role in the mitochondrial apoptotic pathway.

### Superoxide mediates the mitochondrial apoptotic pathway in HUVEC cells, activated by intense heat stress

**Figure 3:**
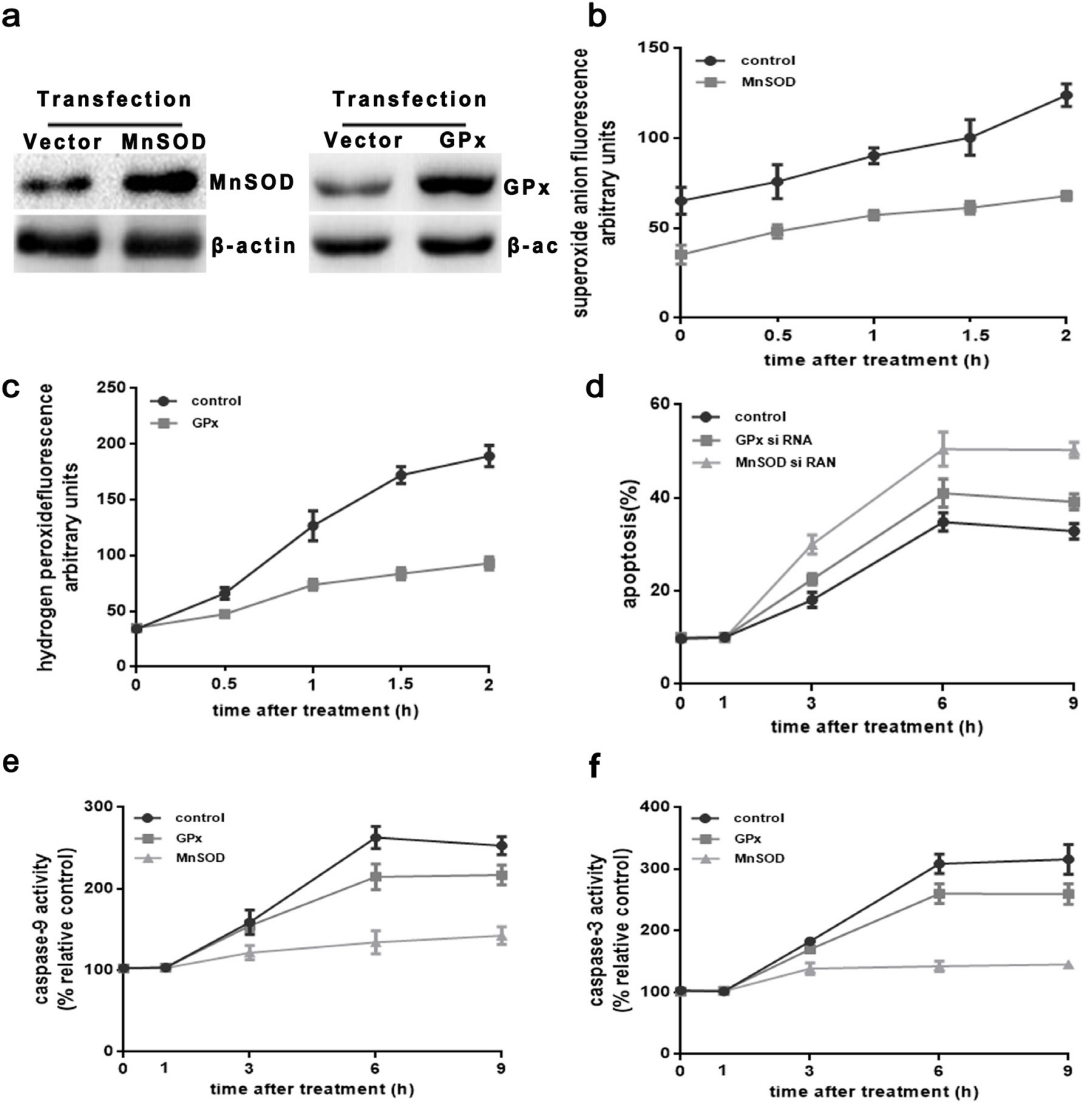
Superoxide mediates induction of the mitochondrial apoptotic pathway in HUVEC cells over-expressing protein MnSOD and GPx. HUVEC cells were stably transfected with or vector control as described in the Materials and Methods. **a**. Western blot analysis of MnSOD and GPx protein expression (cropped) in transfected cells. β-actin served as an internal control. **b**. and **c**. transfected cells were cultured at 43°C for 2h, and incubated at 37°C for different times as indicated (0h, 0.5h, 1h, or 2h). Superoxide anion assay kit and PF6-AM analysis heat stress-induced O_2_^.-^ and H_2_O_2_, respectively. **d-f**. Transfected cells were cultured at 43°C for 2h, then further incubated at 37°C for different times as indicated (0h, 1h, 3h, 6h, or 9h). Apoptosis was analyzed by flow cytometry using Annexin V-FITC/PI staining. Enzymatic activity of caspase-9 and-3 was measured in cell lysates using the fluorogenic substrates Ac-LEHD-AFC and Ac-DEVD-AMC, respectively, and activity was expressed relative to the control at 37°C. Each value represents the mean ± SD of three separate experiments.

**Figure 4:**
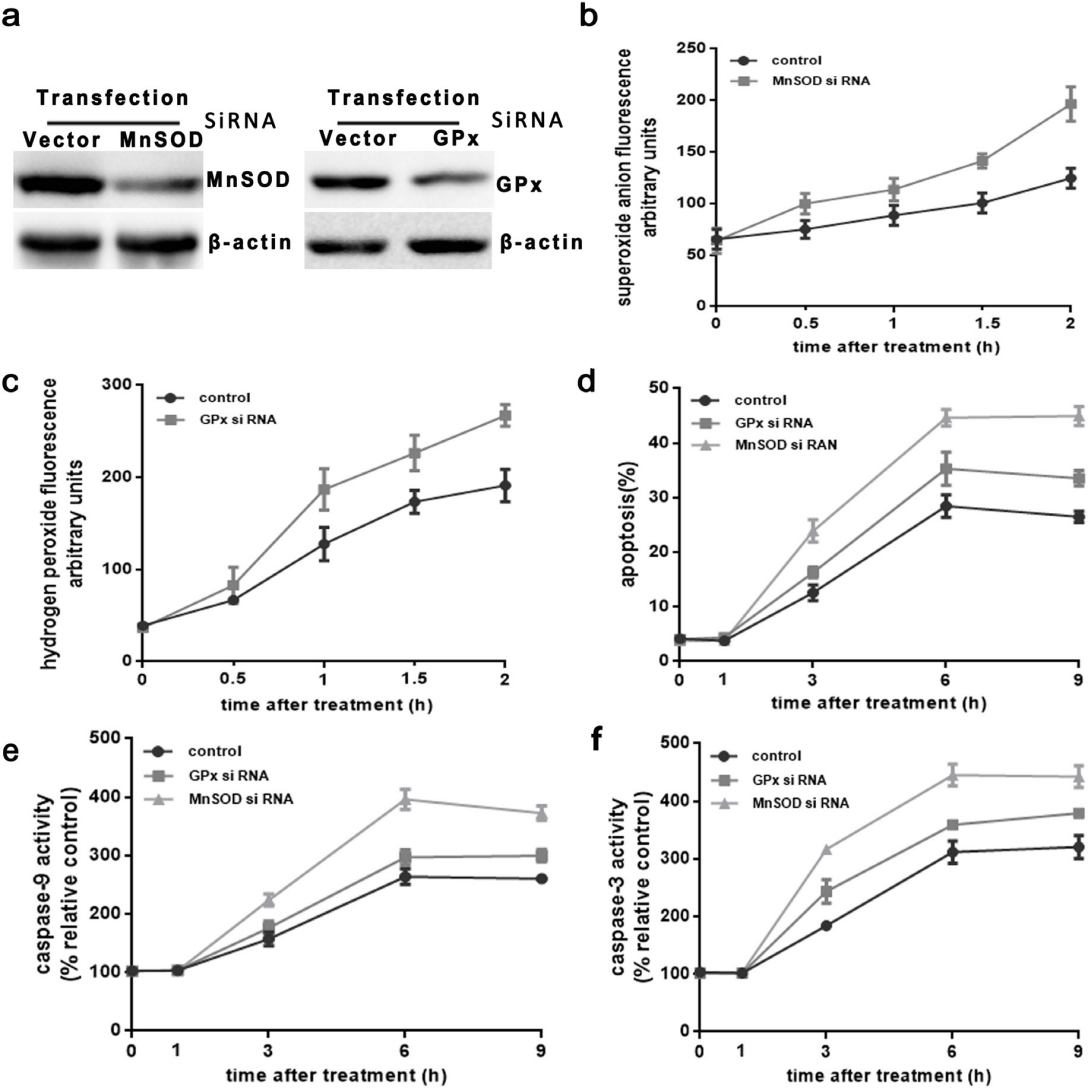
Superoxide mediates induction of the mitochondrial apoptotic pathway in MnSOD and GPx siRNA transfectant HUVEC cells HUVEC cells were stably transfected with scrambledsiRNA(Scr) or MnSOD si RNA and GPx si RNA. **a**. Western blot analysis of MnSOD and GPx protein expression (cropped) in transfected cells. β-actin served as an internal control. **b**. and **c**. transfected cells were cultured at 43°C for 2h, and incubated at 37°C for different times as indicated (0h, 0.5h, 1h, or 2h). Superoxide anion assay kit and PF6-AM analysis heat stress-induced O_2_^.-^ and H_2_O_2_, respectively. **d-f**. Transfected cells were cultured at 43°C for 2h, then further incubated at 37°C for different times as indicated (0h, 1h, 3h, 6h, or 9h). Apoptosis was analyzed by flow cytometry using Annexin V-FITC/PI staining. Enzymatic activity of caspase-9 and-3 was measured in cell lysates using the fluorogenic substrates Ac-LEHD-AFC and Ac-DEVD-AMC, respectively, and activity was expressed relative to the control at 37°C. Each value represents the mean ± SD of three separate experiments.

To further confirm the role of O_2_^.-^ in the mitochondrial apoptotic pathway upon activation by intense heat stress, HUVEC cells were stably transfected with either control empty vector, or plasmids expressing the antioxidant enzymes MnSOD, or GPx, to assess their effects on apoptosis and ROS generation. Over-expression of the enzymes was monitored by western blot. Successfully transfected cells displayed an increase in levels of enzyme expression, as compared to the vector control (Figure 3a). As shown in Figure 3b and 3c, flow cytometric analysis indicated a drastic reduction in heat stress-induced O_2_^.-^ generation in MnSOD-transfected cells, and H_2_O_2_ production in GPx-transfected cells, as compared to control cells, verifying the specificity of heat stress responses in stably-transfected cells. In Figure 3d and 3e, the levels of caspase-9,-3 and apoptosis were both significantly decreased in MnSOD- and GPx-transfected cells as compared to vector control cells. The effect was most prominent in cells over-expressing MnSOD, which can continue to restrain apoptosis through the mitochondrial apoptotic pathway for up to 9 h after intense heat stress. The effect described above are reversed when down-regulating the expression of MnSOD (Figure 4). These results suggested to us that although H_2_O_2_ is involved in heat stress-induced mitochondrial apoptotic pathway, mitochondrial superoxide may be the major regulator of heat stress-mediated induction of the mitochondrial apoptotic pathway.

### Intense heat stress regulates Bcl-2 through superoxide in HUVEC cells

**Figure 5:**
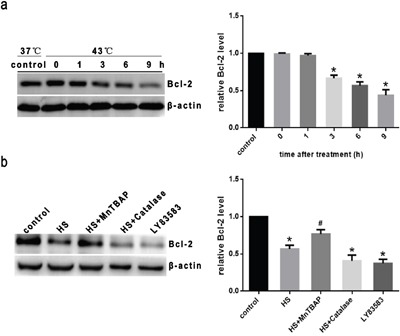
Intense heat stress regulates Bcl-2 through superoxide in HUVEC cells **a**. Cells were cultured at 43°C for 2h, then incubated at 37°C for different lengthes of time as indicated (0h, 1h, 3h, 6h or 9h). **b**. Cells were treated with 100μM MnTBAP or 1000 U/μl catalase for 0.5h prior to heat stress at 43°C for 2h, and further incubated at 37°C for 6h. 10μM LY83583 was used as positive control. Western blot analysis of Bcl-2 protein expression in HUVEC cells. Each value represents the mean ± SD of three separate experiments, *P < 0.05, compared to control group (37°C), ^#^*P* < 0.05, compared to heat stress group (43°C).

To explore the mechanisms of regulation of intense heat stress-induced apoptosis, we tested the expression levels of Bcl-2, a key anti-apoptotic protein involved in the mitochondrial death pathway. As shown in Figure 5a, the expression of Bcl-2 decreased in a time - dependent manner after exposure to intense heat stress, which was consistent with the time period during which caspase-9,-3 was activated and apoptosis occurred. In previous studies, we have confirmed that over-expression Bcl-2 in HUVEC cells significantly decreases intense heat stress-induced apoptosis via the mitochondrial apoptotic pathway [10, 12]. To determine the role of superoxide, cells were treated with intense heat stress in the presence or absence MnTBAP and catalase, then analyzed for Bcl-2 levels by western blotting. The expression of Bcl-2 was decreased significantly by LY83583 (O_2_^.-^ donor), and intense heat stress-induced down-regulation of Bcl-2 was inhibited by MnTBAP, but not by catalase (Figure 5b). These results indicated that the anti-apoptotic protein Bcl-2 negatively regulates the mitochondrial apoptotic pathway upon intense heat stress, and that superoxide might be a key mediator of intense heat stress-induced Bcl-2 down-regulation.

### Intense heat stress induces Bcl-2 ubiquitination through superoxide in HUVEC cells

**Figure 6:**
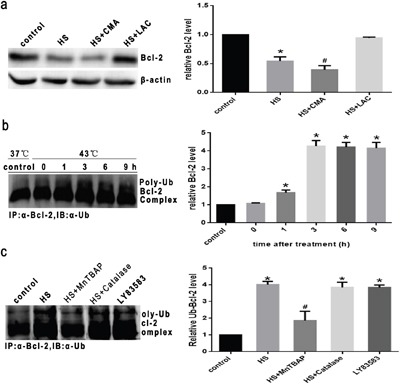
Intense heat stress induces Bcl-2 ubiquitination via superoxide activity in HUVEC cells **a**. Cells were pretreated with the proteasome inhibitor lactacystin (LAC, 10μM) or lysosome inhibitor concanamycin A (CMA, 1μM) for 1h prior to heat stress at 43°C for 2h, and further incubated at 37°C for 6h. Western blot analysis of Bcl-2 protein expression. **b**. Cells were pretreated with LAC (10μM) for 1 h to prevent proteasomal degradation of Bcl-2, then cultured at 43°C for 2h, and incubated at 37°C for different lengths of times as indicated (0h, 1h, 3h, 6h or 9h). **c**. Cells were pretreated with LAC (10μM) for 1h, and in the presence or absence of MnTBAP (100μM) or Catalase (1000 U/μl) for 0.5h prior to heat stress (43°C) for 2h, and further incubated at 37°C for 6h. LY83583 (10μM) was used as positive control. Cell lysates were immunoprecipitated with anti-Bcl-2 antibody and the immune complexes were analyzed for ubiquitin by Western blotting. Each value represents the mean ± SD of three separate experiments, *P < 0.05, compared to control group (37°C), ^#^*P* < 0.05, compared to heat stress group (43°C).

Ubiquitination is a major cellular process for selective targeting and removal of innumerable cellular proteins through proteasomal degradation. Accumulating evidence indicates that the anti-apoptotic protein Bcl-2 is down regulated primarily via the proteasomal degradation pathway under diverse apoptotic conditions [13, 15, 17]. To investigate whether this pathway is involved in down regulation of Bcl-2 induced by heat stress, HUVEC cells were treated in the presence or absence of lactacystin (LAC, a highly specific proteasome inhibitor) prior to heat stress at 43°C. Since lysosomal degradation is another pathway involved in protein degradation, HUVEC cells were also observed in the presence or absence of concanamycin A (CMA), a highly specific lysosome inhibitor, prior to heat stress, and then analyzed for Bcl-2 by western blotting. As shown in Figure 6a, LAC, but not CMA, completely inhibited Bcl-2 down regulation after intense heat stress, suggesting that proteasomal degradation may be the primary mechanism of Bcl-2 dampening upon heat stress. To explore whether heat stress can induce Bcl-2 ubiquitination in HUVEC cells, cells were pretreated with 10μM LAC for 1 hr to prevent proteasomal degradation of Bcl-2, followed by culturing at 43°C for 2h, further incubated at 37°C for different times as indicated (0h, 1h, 3h, 6h or 9h). Cell lysates were then immunoprecipitated with an anti-Bcl-2 antibody, and the immune complexes were analyzed for ubiquitin by Western blotting. As shown in Figure 6b, Bcl-2 ubiquitination was drastically incraesed after 3h of heat stress, and this increase continued to 9h. As shown in Figure 6c, treatment of cells with MnTBAP completely inhibited the ubiquitination of Bcl-2 induced by heat stress, whereas catalase was unable to inhibit ubiquitination, and LY83583 (O_2_^.-^ donor) also induced Bcl-2 ubiquitination. These results indicate that superoxide is the major oxidative species involved in heat stress-induced Bcl-2 degradation via the ubiquitination pathway.

### Superoxide mediates dephosphorylation of Bcl-2 and ERK1/2 induced by intense heat stress in HUVEC cells

**Figure 7:**
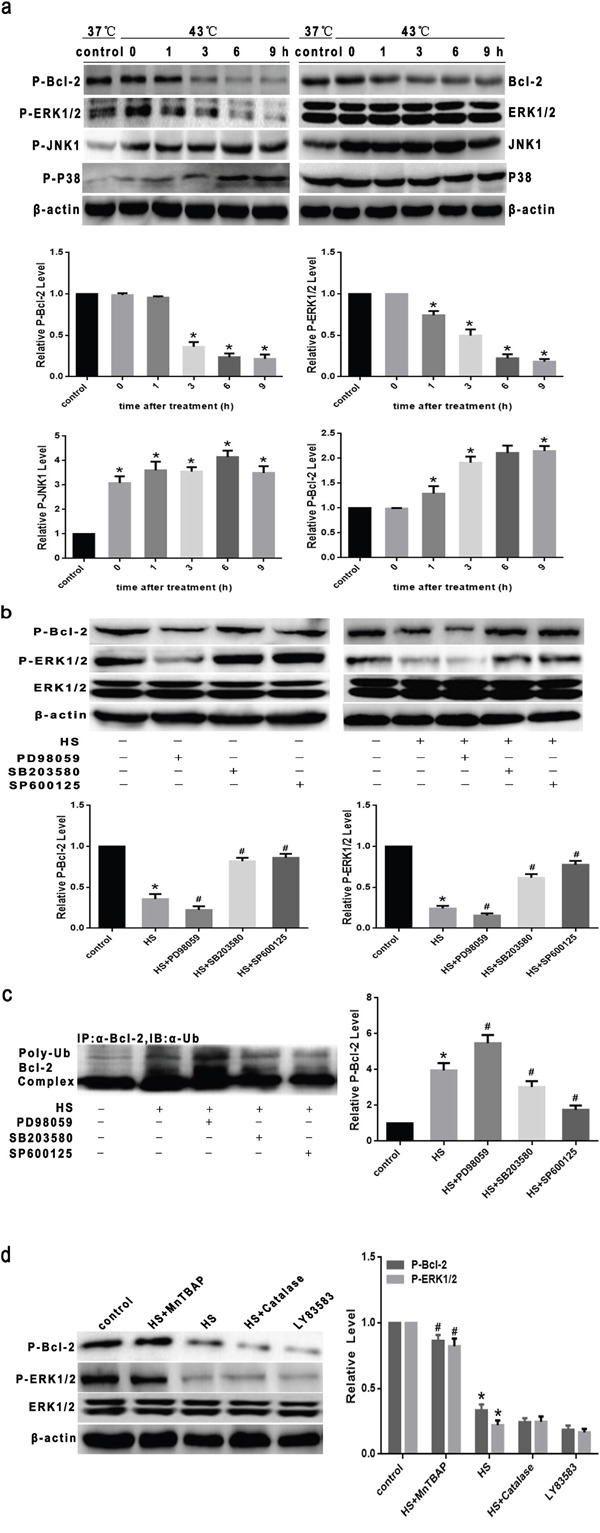
Superoxide mediates dephosphorylation of Bcl-2 and ERK1/2 induced by intense heat stress in HUVEC cells **a**. Cells were cultured at 43°C for 2h, then incubated at 37°C for the indicated times (0h, 1h, 3h, 6h or 9h). Expression levels of phospho-Bcl-2, phosho-ERK1/2 (p-ERK1/2), phospho-JNK1 (p-JNK1) and phospho-p38 kinase (p-p38) were analyzed by Western blotting. Blots were re-probed with total Bcl-2, ERK1/2, JNK1, p38 and β-actin antibody to confirm equal loading of the samples. **b**. Cells were incubated in the presence or absence of PD98059 (ERK1/2 inhibitor, 25μM), SB203580 (p38 inhibitor, 10μM), or SP600125 (JNK inhibitor, 10 μM), then cultured at 37°C or 43°C for 2h, further incubated at 37°C for 6h. P-Bcl-2 and p-ERK1/2 levels were analyzed by Western blotting. Blots were re-probed with totalERK1/2 and β-actin antibodies to confirm equal loading of the samples. **c**. Cells were incubated in the presence or absence of PD98059 (25μM), SB203580 10μM), and SP600125 (10 μM), then cultured at 43°C for 2h, further incubated at 37°C for 6h. Cell lysates were immunoprecipitated with an anti-Bcl-2 antibody and immune complexes were analyzed for antibodies by Western blotting. **d**. Cells were incubated in the presence or absence of MnTBAP (100μM) or catalase (1000 U/μl) for 0.5h prior to heat stress at 43°C for 2hrs, and further incubated at 37°C for 6hrs. LY83583 (10μM) was used as positive control. P-Bcl-2 and p-ERK1/2 were analyzed by Western blotting. Blots were re-probed with total ERK1/2 and β-actin antibodies to confirm equal loading of the samples. Each value represents the mean ± SD of three separate experiments, *P < 0.05, relative to the control group (37°C ), ^#^ *P* < 0.05, as compared to the heat stress group (43°C).

It has been reported that Bcl-2 ubiquitination is regulated by its phosphorylation status [14, 20, 21]. However, the precise mechanisms of regulation are unclear. Agents that stimulate apoptosis have been shown to induce dephosphorylation of Bcl-2 which may trigger ubiquitination and subsequent proteasomal degradation [16, 22]. In the present study, we probed whether heat stress can induce dephosphorylation of Bcl-2 and what mechanisms are involved. As shown in Figure 7a, Bcl-2 dephosphorylation was induced at 3h after intense heat stress, and continued to 9h, which is also consistent with the time period during which there is caspase-9,-3 activation Bcl-2 ubiquitination and apoptosis. To explore whether mitogen-activated protein (MAP) kinases, modulators of major phosphorylation - dephosphorylation signaling cascades, are responsible for Bcl-2 dephosphorylation and ubiquitination in response to intense heat stress, we assessed their expression levels. Cells were cultured at 43°C for 2h, further incubated at 37°C for various lengths of time as indicated (0h, 1h, 3h, 6h or 9h), and then lysed for western blot analysis of MAP kinase activation, specifically of ERK1/2, JNK1 and p-38 kinases. ERK1/2 inactivation (dephosphorylation) was detected as early as 1hr after heat stress, prior to the dephosphorylation of Bcl-2. JNK1 and p38 kinases displayed an opposite trend of increasing phosphorylation in a time-dependent manner after heat stress. These results indicate that ERK1/2, not JNK1 or p38 kinase, is the potential Bcl-2 kinase which acts upstream of Bcl-2 dephosphorylation after heat stress in HUVEC cells.

To confirm the role of ERK1/2 in phosphorylation and ubiquitination of Bcl-2, cells were pretreated in the presence or absence of MAP kinase inhibitors PD98059 (ERK1/2 inhibitor), SB203580 (p38 inhibitor), or SP600125 (JNK inhibitor). As shown in Figure 7b and 7c, the ERK1/2 inhibitor PD98059 led to increased levels Bcl-2 dephosphorylation and ubiquitination after heat stress, whereas the JNK and p38 inhibitors did not affect dephosphorylation and ubiquitination. These results provide further supporting evidence that ERK1/2 plays an important role in Bcl-2 phosphorylation, and that its inactivation uopn heat stress contributes to Bcl-2 ubiquitination and degradation.

Building on our findings described above, that Bcl-2 ubiquitination was mediated by superoxide after heat stress, we investigated whether dephosphorylation of ERK1/2 and Bcl-2 is dependent on superoxide. Cells were pretreated in the presence or absence of MnTBAP or catalase. As shown in Figure 7d, treatment of cells with MnTBAP completely inhibited the dephosphorylation of ERK1/2 and Bcl-2 after heat stress, catalase had no effect, and LY83583 (O_2_^.-^ donor) induced dephosphorylation of ERK1/2 and Bcl-2. Together, these results suggest that superoxide mediates dephosphorylation and ubiquitin–proteasomal degradation of Bcl-2 by inactivation of ERK1/2 after heat stress in HUVEC cells.

## DISCUSSION

**Figure 8:**
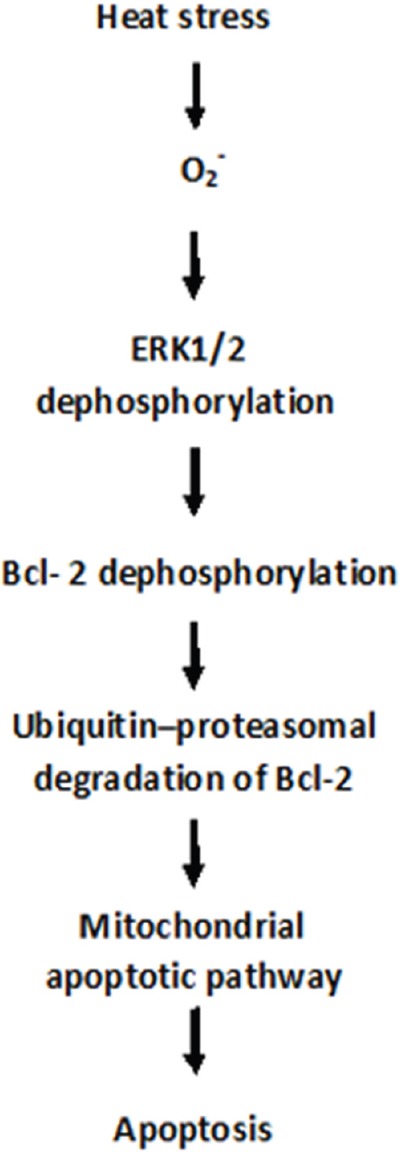
Proposed redox-mediated down-regulation of Bcl-2 and apoptosis induce by heat stress in HUVEC cells Our study provides evidence that superoxide may be a key mediator of heat stress-induced apoptosis through a mechanism related to Bcl-2 down-regulation and activation of the mitochondrial apoptotic pathway in HUVEC cells. Superoxide appears to act as an upstream signaling molecule involved in Bcl-2 down-regulation via ubiquitin–proteasomal degradation that is triggered by inactivation of ERK1/2.

Heat is a significant extracellular stimulus that can result in cell toxicity [4]. Several studies have concluded that endothelial cells may be an early target in heat stress-mediated tissue injury [10]. Our previous work showed that heat stress induces apoptosis in HUVEC cells [10–12], but the underlying mechanism remained to be investigated. In this study, we found that intense heat stress induced the mitochondrial apoptotic pathway through ROS generation, which mediated dephosphorylation and ubiquitin–proteasomal degradation of Bcl-2 by inactivation of ERK1/2 in HUVEC cells (Figure 8).

Reactive oxygen species (ROS) include partially reduced oxygen metabolites such as superoxide (O_2_^.-^), hydrogen peroxide (H_2_O_2_) and hydroxyl radical (OH). The release of these species may increase upon exposure to cytotoxic agents (e.g. irradiation, pesticides, environmental pollutants, and anticancer drugs [23]. Toxicity and cell damage induced by ROS can be scavenged and repaired by non-enzymatic (e.g. vitamin E, glutathione, ascorbate, and carotenoids) and enzymatic antioxidants including superoxide dismutase (SOD), glutathione peroxidase (Gpx) and catalase. Additionally, superoxide radicals produced by the mitochondrial respiratory chain can be dismutase by mitochondrial SOD (MnSOD)[11, 23–26]. Previous studies have confirmed that heat stress -induced ROS generation may act in concert to promote cellular apoptosis [10, 11, 23]. However, the identity of the specific ROS involved in the process remained unclear and controversial. In this study, we found that intense heat stress induced production of both O_2_^.-^ and H_2_O_2_ reactive species, however, O_2_^.-^ noticeably increased immediately after heat stress (0h), while H_2_O_2_ levels increase significantly only 0.5h after heat stress. Although treatment with free radical scavengers of both species inhibited activation of caspase-9,-3 and apoptosis after intense heat stress (6h), the potent inhibitory effect of MnTBAP further indicates that superoxide plays a major role in the mitochondrial apoptotic pathway. We also found that production of mitochondrial superoxide displayed a similar increasing trend in the generation of O_2_^.-^ after heat stress. We further confirmed the specific ROS involved in apoptosis upon intense heat stress by assaying HUVEC cells stably transfected with MnSOD or GPx. Our results demonstrated that MnSOD effectively inhibits caspase-9,-3 and apoptosis, indicating that mitochondrial superoxide, but not H_2_O_2_, may be the primary oxidative species and key regulator of heat stress-mediated induction of the mitochondrial apoptotic pathway.

Several studies illustrate that Bcl-2 is a key regulatory component of the mitochondrial death pathway [15, 27]. The anti-apoptotic function of Bcl-2 depends on its expression levels, which may be mediated by various mechanisms including phosphorylation, degradation, transcription, and posttranslational modification [17, 28]. So far the purpose of Bcl-2 phosphorylation /dephosphorylation remained unclear and controversial; for example, it has been suggested that Bcl-2 phosphorylation is involved in the inactivation of anti-apoptotic function, and promotes anti-apoptotic mechanisms [29, 30]. Bcl-2 dephosphorylation promotes cell death through mediating ubiquitin-dependent degradation. It is likely that the regulation of the anti-apoptotic function of Bcl-2 by phosphorylation /dephosphorylation is dependent on cell-type specific factors that are as yet undefined. The ubiquitin-proteasomal pathway is the primary mechanism of Bcl-2 degradation, which plays a critical role in apoptosis induction by various stimuli [13, 31]. It has also been well documented that the expression and stability levels of the Bcl-2 protein can be mediated by different ROS, through various mechanisms [13, 16, 17]. In our current study, we have conclusively shown that intense heat stress induces down-regulation of Bcl-2 in a time - dependent manner, which is concurrent with activation of caspase-9,-3 and induction of apoptosis. We also observed that heat stress induced down-regulation of Bcl-2 via ubiquitin–proteasomal degradation. These observations indicate that the dephosphorylation of Bcl-2 results in ubiquitin-dependent degradation, then subsequent inactivation of anti-apoptotic function. To further probe the mechanisms involved, we determined the effect of various ROS modulators on ubiquitination of Bcl-2. We found that the superoxide scavenger MnTBAP inhibited ubiquitination of Bcl-2, the H_2_O_2_ scavenger catalase had no effect after heat stress, and LY83583 (O_2_^.-^ donor) also induced Bcl-2 ubiquitination. Together the above results suggest that superoxide may be the major ROS mediating Bcl-2 ubiquitination after heat stress in HUVEC cells.

Mitogen-activated protein (MAP) kinases is also known as a vital regulator of diverse cellular functions, such as cell proliferation, apoptosis, migration and differentiation [32]. At the cellular level, inhibiting phosphorylation of d ERK1/2 induces cellular apoptosis [33]. Meanwhile, activating ERK1/2 regulates positively cell proliferation [34]. These observations indicate that ERK1/2 plays dual roles in cell death and survival. It has been reported that ERK1/2 implicated in the dephosphorylation and ubiquitination of Bcl-2 [17, 35, 36]. We thus tested whether heat stress induces Bcl-2 dephosphorylation, and probed the potential mechanism between Bcl-2 dephosphorylation and MAP kinase signaling cascades. Our results indicate that Bcl-2 dephosphorylation and ERK1/2 occur upstream of Bcl-2 dephosphorylation after exposure to intense heat stress. To obtain further evidence confirming the role of ERK1/2, cells were pretreated in the presence or absence of MAP kinase inhibitors, and it was determined that only the ERK1/2 inhibitor PD98059 increases Bcl-2 dephosphorylation and ubiquitination, and inhibitors of JNK and p38 kinases have no effect on this process. We also observed that Bcl-2 ubiquitin-mediated degradation is dependent on ERK1/2 and Bcl-2 dephosphorylation. Many studies indicate that ROS are upstream mediators of ERK dephosphorylation. According to Liu and Chang [39] and Kim et al. [40], ROS caused a significant inhibition of ERK1/2. Zhang et al. have also reported that ROS inhibit ERK phosphorylation in Hep-2 cells after 9-hydroxypheophorbide α-mediated photodynamic therapy [41]. Our findings suggest that this effect can be prevented by superoxide scavenging by MnTBAP but not by the H_2_O_2_ scavenger catalase, further verifying the role of superoxide, in dephosphorylation and ubiquitin–proteasomal degradation of Bcl-2 via the MAP kinase ERK1/2 pathway after heat stress in HUVEC cells. Thus, the present findings are consistent with those of previous reports.

In conclusion, our study provides evidence that superoxide may be a key mediator of heat stress-induced apoptosis through a mechanism related to Bcl-2 down-regulation and activation of the mitochondrial apoptotic pathway in HUVEC cells. Superoxide appears to act as an upstream signaling molecule involved in Bcl-2 down-regulation via ubiquitin–proteasomal degradation that is triggered by inactivation of ERK1/2. However, it remains to be determined whether similar regulatory mechanisms are in place *in vivo* and in other cellular systems.

## MATERIALS AND METHODS

### Cell culture, heat treatment and cell viability assays

Human umbilical vein endothelial cells (HUVECs) were purchased from the Shanghai Institute of Cell Biology, Chinese Academy of Sciences. Cells were grown in culture medium as recommended by the manufacturer, and used at passage 3. Cell culture dishes containing HUVEC cells were sealed with Parafilm and immersed for 2 h in a circulating water bath thermo-regulated at 37°C±0.5°C (control) or at 39°C, 41°C, 43°C, or 45°C±0.5°C (heat stress) [10, 11, 42]. Culture medium was then replaced with fresh medium and the cells were further incubated at 37°C for different time periods, as indicated. Cell proliferation was assessed by using the cell counting kit 8 (CCK-8; Dojindo, Kumamoto, Japan) method according to the manufacturer's instructions.

### Apoptosis assay

For cell cycle analysis, cells were either kept untreated or exposed to 43°C for 2 h before being analyzed by flow cytometry. The detection was performed according to the Annexin V-FITC apoptosis detection kit manual (Invitrogen). About 1×10^6^ cells were collected, washed with ice-cold PBS, and resuspended in binding buffer containing Annexin V-FITC. After 10 min incubation in the dark at room temperature, buffer was removed by centrifugation. Cells were resuspended in reaction buffer containing propidium iodide (PI), and then immediately subject to flow cytometric analysis to detect apoptosis.

### Measurement of superoxide anion production

HUVEC cells were heat stressed at 43°C for 2h, and then further incubated at 37°C for 0, 0.5, 1, or 2 h. O_2_^.-^ levels were detected using a commercial superoxide anion assay kit (Sigma Aldrich Co.) according to the manufacturer's instructions. This measurement is based on the oxidation of luminol by O_2_^.-^ resulting in the formation of chemiluminescence light. HUVEC cells were incubated with luminol solution and enhancer solution, and the luminescence intensity was read every 10 min during a 4 h-period.

### Measurement of hydrogen peroxide production

HUVEC cells were heat stressed at 43°C for 2h, and then further incubated at 37°C for 0, 0.5, 1, or 2 h. Intracellular H2O2 levels were determined by fluorescent probe peroxyfluor-6 acetoxymethyl ester (PF6-AM) [43]. HUVEC cells were incubated with 5 M PF6-AM in Hanks’ balanced salt solution (HBSS) containing 20 mM HEPES (HBSS-H) for 30 min at 37°C. The fluorescence intensity of PF6-AM probes was measured in a luminometer (Berthold-Biolumat LB937).

### Analysis of ONOO^-^ production

ONOO^-^ was measured by luminol-amplified chemiluminescence as previously described [44]. The reaction mixture (total volume 1 mL) consisted of: HBSS-EDTA (1 mM); microsomes (50 μg of protein); L-arginine (100 μM); NADPH (100 μM); FAD (5 μM); FMN(5 μM); tetrahydrobiopterin (5 μM); calmodulin (1 PM) and 2.5, μM scopoletin. HUVEC cells were incubated with compound at 37°C for 10min, then the luminescence intensity was measured in a luminometer.

### Detection of mitochondrial superoxide

To analyze the kinetics of mitochondrial superoxide generation, HUVEC cells were heat stressed for 2h at 43°C, and incubated at 37°C for 0, 0.5, 1, or 2 h. Mitochondrial superoxide formation was examined by fluorescence microscopy using MitoSOXTM Red as the specific fluorescent probe. Briefly, cells were incubated with 5μM of the probe for 30 min at 37°C in the dark. They were then washed thoroughly with warm HBSS buffer and mounted for imaging. MitoSOXTM Red was visualized using an excitation wavelength of 546 nm and an emitter band pass of 605 nm.

### Caspase activity assay

After exposure to 43°C heat stress for 2h, cells were further incubated at 37°C for the indicated times. Cells were harvested and lysed, and lysates were incubated at -80°C for 30 min prior to incubation with the appropriate caspase substrates at 37°C. Caspase activity were measured by monitoring cleavage of the following fluorogenic peptide substrates [23, 45]: Ac-LEHD-AFC, caspase-9; Ac-DEVD-AMC, caspase-3, using a Quadruple Monochromator Microplate Reader (Infinite M1000, Tecan US, NC, USA). Caspase activity is represented as cumulative fluorescence of the kinetic reaction relative to untreated controls.

### Plasmid construction and stable transfection

The eukaryotic expression vectors pcDNA3.1-MnSOD and pcDNA3.1- GPx for MnSOD and GPx were constructed by Shanghai Genechem (Genechem Incorporation, Shanghai, China). HUVEC cells were transfected with empty vector (pcDNA3.1), pcDNA3.1-MnSOD or pcDNA3.1- GPx using Lipofectamine 2000. Culture medium containing G418 was used to select stable transfectants.

### Western blot analysis

Cells were pretreated with or without heat stress at 43°C for 2h, and further incubated at 37°C for different time as indicated. Western blot analysis was performed as described previously [45], using the following antibodies:Bcl-2, Apaf-1, phospho-ERK1/2(PT185+Y187), ERK1/2, Phospho-JNK1(PT185+Y187), JNK1, phospho-P38(PT180+Y182), P38 (all used at 1:1000; Cell Signaling Technology, Danvers, USA). A HRP-conjugated anti-rabbit IgG antibody was used as the secondary antibody (Zhongshan Inc, China), and signal was visualized enhanced chemiluminescence (Pierce, Rockford, IL, USA).

### Immunoprecipitation

Cells were pretreated with or without heat stress at 43°C for 2h, and further incubated at 37°C for different times, as indicated. Immunoprecipitation analysis was performed as described previously [15, 17]: cell lysates were incubated withanti-Bcl-2 antibody at 4°C for 14 h, followed by incubation with protein G-conjugated agarose for 4 hat 4 °C. Immune complexes were separated by 10% SDS-polyacrylamide gel electrophoresis and analyzed by Western blot as described above.

### Statistical analysis

All data were analyzed for statistical significance using SPSS 13.0 software (SPSS, Chicago, IL, USA). Data were expressed as mean ± SD from at least 3 independent experiments performed in duplicate. Statistical comparisons of the results were performed using one -way analysis of variance (ANOVA). P value <0.05 was considered to be statistically significant.
